# Port site metastasis following diagnostic laparoscopy for a malignant Gastro-intestinal stromal tumour

**DOI:** 10.1186/1477-7819-6-55

**Published:** 2008-05-24

**Authors:** Andrew R Davies, Waseem Ahmed, Shaun F Purkiss

**Affiliations:** 1 Department. of Surgery, Whipps Cross University Hospital, Leytonstone, London, UK

## Abstract

**Background:**

Gastro-Intestinal stromal tumours (GISTs) are rare and our understanding of their natural history and optimal treatment are continually evolving. Port site metastasis after laparoscopy for a GIST is an extremely rare phenomenon.

**Case presentation:**

We report a case with relevant imaging and discuss factors that may have contributed to the development of this isolated metastasis.

**Conclusion:**

Percutaneous methods of sampling GIST tumours for analysis should be avoided if at all possible. When necessary, prophylactic measures should be utilised to minimise the risk of seeding.

## Background

Gastro-Intestinal stromal tumors (GISTs) are rare, and our understanding of their natural history and optimal treatment are continually evolving. This case report of a port site metastasis following laparoscopy for a malignant GIST is, to our knowledge, only the second documented case. As well as an outline of the case itself, the question as to why these arise and what the optimal treatment should be is also discussed.

## Case presentation

A 75 year old female presents to her General Practitioner with symptoms of lethargy, weight loss and occasional vomiting. Abdominal examination reveals a large mass in the epigastrium and blood investigations reveal a microcytic anaemia with a Haemoglobin of 9.4 g/dL. Her only past medical history includes atrial fibrillation and a total abdominal hysterectomy and bilateral salpingo-oophorectomy for benign disease.

**Figure 1:**
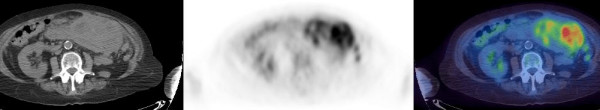
Advanced tumour with uptake seen within a large mass and throughout the peritoneal cavity.

She is urgently referred for further investigation. Computed Tomography (CT) scanning demonstrates a 13 cm multi-loculated mass adjacent to the anterior wall of the stomach radiologically suspicious for a Gastro-intestinal stromal tumour (GIST). A subsequent PET-CT scan also demonstrated multiple peritoneal seedlings (Figure 1). Upper Gastro-intestinal Endoscopy showed extrinsic compression of anterior gastric wall, with possible communication to a pus filled cavity. Unfortunately, multiple biopsies failed to yield a histological diagnosis.

At diagnostic laparoscopy (performed to obtain tissue diagnosis and assess potential tumour stage), the large mass anterior to the stomach was biopsied as were the peritoneal seedlings. Histology demonstrated a malignant GIST, strongly positive for CD117. She was discussed at the regional Upper GI Multi-Disciplinary meeting, and the decision made to commence systemic oncological therapy with Glivec (Imatinib).

**Figure 2:**
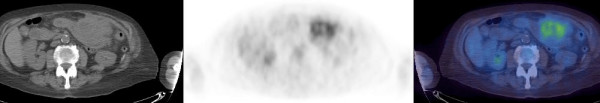
Partial Response with significantly reduced uptake on PET.

**Figure 3:**
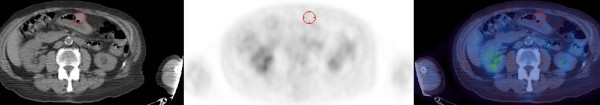
No residual tumour. No pathological uptake seen within the abdominal cavity.

Encouragingly, her response to treatment was excellent and within one month a demonstrable radiological improvement was noted (Figure 2). At ten months there was no evidence of residual disease on PET-CT (Figure 3). As per unit procedure, she subsequently entered a routine follow up protocol which included regular clinical and radiological review.

**Figure 4:**
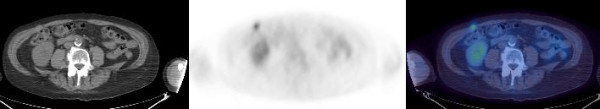
Port site metastasis. Uptake can be clearly seen in the Abdominal wall (Internal oblique) corresponding to the site of the laparoscopic port.

Unfortunately, although she remains clinically asymptomatic, a follow up PET-CT (approximately 22 months from initial diagnosis and commencement of treatment) demonstrated a small discrete focus of uptake within the internal oblique muscle on the right side (Figure 4). This correlates with the location of the port site used at the time of diagnostic laparoscopy.

In light of the confirmation of a port site metastasis (PSM), she is due to be discussed again in the MDT meeting prior to making a final decision regarding further treatment. The evidence base for the treatment of port site recurrences originating from common tumours is limited. In the context of this particular case i.e. a GI Stromal tumour, it is non-existent.

## Discussion

Gastro-intestinal stromal tumours are the most common mesenchymal tumour affecting the GI tract. They may present in a variety of ways, can range from small to very large and demonstrate a great diversity in their malignant potential. Tumours are often positive for the c-KIT (CD117) protein which is a tyrosine kinase receptor and is the most sensitive and specific marker. Mutations of this CD117 proto-oncogene are associated with stromal tumours and hence the use of long term Imatinib (a CD117 inhibitor) in their treatment [1].

In many ways, the presentation in this case with a malignant GIST is fairly typical. Bleeding, and resultant anaemia, is often the precipitating event leading up to presentation. Malignant tumours often have a palpable mass at diagnosis and response to Glivec (although somewhat variable) can be dramatic.

Generally speaking, pre-operative biopsy is not advised in patients who are radiologically diagnosed with a GIST and resectable. However, in the event of more widespread disease, where histological confirmation is required to commence oncological treatment, endoscopic biopsy techniques are preferred in order to limit the possibility of peritoneal contamination. Failing this, percutaneous techniques (either radiologically guided or via laparoscopy) remain the only alternative, both presenting a theoretical risk of tumour seeding [2,3].

Port Site metastases (PSM) although rare, have been extensively documented for other gynaecological and GI malignancies. When they occur, they often do so in the presence of advanced disease, and it is not uncommon for them to occur in isolation [4,5]. Their presence is usually associated with poor long term outcomes.

Mechanisms thought to be involved in the development of PSMs include high pressure CO2 insufflation, the degree of tumour manipulation during surgery, direct seeding during unprotected tumour/instrument withdrawal as well as the biological characteristics of the cancer itself [6,7].

In this particular case, laparoscopy (and hence gas insufflation) was required because of failure of alternative techniques to establish the diagnosis. The tumour was biopsied and the biopsy instrument removed through the port (in theory protecting the specimen from contacting the layers of the abdominal wall), although the port itself then required removal. The advanced nature of the tumour also increased the risk of a PSM but as far as we know, the port did not enter the peritoneal cavity at the site of an existing peritoneal metastasis.

As far as the authors are aware, there is only one previously documented case of port site metastasis (PSM) following laparoscopy for a stromal tumour [8]. Although an extremely rare phenomenon, the implication is that prophylactic measures of preventing seeding should be undertaken when performing laparoscopy for stromal tumours. Such measures could include the use of protective retrieval bags or peritoneal lavage with anti-adhesive or cytocidal solutions, although evidence for the latter is limited to say the least. Where possible, the use of any percutaneous technique to obtain diagnosis should be avoided.

In this particular case, further treatment options could include surgical resection of the port site or the use of a second line agent such as sunitinib. This latter decision would be made on the assumption that the development of a port site metastasis whilst on first line treatment, confers the development of tumour resistance to this intial therapy. This is a phenomenon that has been increasingly well documented in the literature [1], and successfully treated with further medical therapy.

## Conclusion

Percutaneous methods of obtaining histological evidence of GIST tumours, should be avoided if at all possible. When necessary, the reporting of PSM's suggests that prophylactic measures should be utilised to minimise the risk of seeding.

## Competing interests

The authors declare that they have no competing interests

## Authors' contributions

AD performed the literature search, wrote and submitted the manuscript

WA assisted with the literature search and obtained the images presented from the Nuclear medicine Dept.

SP performed the surgery, was the consultant in charge of the patients' care and made alterations to the final draft of the paper.

All authors read and approved final manuscript

## Consent

Written consent was obtained from the patient for publication of this case report
